# Metabolic Dysfunction–Associated Fatty Liver Disease (MAFLD) and Thyroid Function in Childhood Obesity: A Vicious Circle?

**DOI:** 10.3390/children11020244

**Published:** 2024-02-15

**Authors:** Valeria Calcaterra, Irene Degrassi, Silvia Taranto, Cecilia Porro, Alice Bianchi, Sara L’assainato, Giustino Simone Silvestro, Antonia Quatrale, Gianvincenzo Zuccotti

**Affiliations:** 1 Pediatric and Adolescent Unit, Department of Internal Medicine, University of Pavia, 27100 Pavia, Italy; 2 Pediatric Department, Buzzi Children’s Hospital, 20154 Milan, Italy; irene.degrassi@asst-fbf-sacco.it (I.D.); silvia.taranto@unimi.it (S.T.); cecilia.porro@unimi.it (C.P.); alice.bianchi1@unimi.it (A.B.); sara.lassainato@unimi.it (S.L.); giustino.silvestro@unimi.it (G.S.S.); antonia.quatrale@unimi.it (A.Q.); gianvincenzo.zuccotti@unimi.it (G.Z.); 3 Department of Biomedical and Clinical Science “L. Sacco”, University of Milan, 20157 Milan, Italy

**Keywords:** metabolic dysfunction-associated fatty liver disease, thyroid function, children, obesity, non-alcoholic fatty liver disease

## Abstract

Metabolic dysfunction–associated fatty liver disease (MAFLD) is a multisystem disorder characterized by the presence of fatty liver degeneration associated with excess adiposity or prediabetes/type 2 diabetes or metabolic dysregulation. An intricate relationship between the liver and thyroid has been reported in both health and disease. Simultaneously, there is a strong correlation between obesity and both MAFLD and thyroid dysfunction. In this narrative review, we highlighted the relationship between MAFLD and thyroid function in children and adolescents with obesity in order to explore how thyroid hormones (THs) act as predisposing factors in the onset, progression, and sustainability of MAFLD. THs are integral to the intricate balance of metabolic activities, ensuring energy homeostasis, and are indispensable for growth and development. Regarding liver homeostasis, THs have been suggested to interact with liver lipid homeostasis through a series of processes, including stimulating the entry of free fatty acids into the liver for esterification into triglycerides and increasing mitochondrial β-oxidation of fatty acids to impact hepatic lipid accumulation. The literature supports a correlation between MAFLD and obesity, THs and obesity, and MAFLD and THs; however, results in the pediatric population are very limited. Even though the underlying pathogenic mechanism involved in the relationship between MAFLD and thyroid function remains not fully elucidated, the role of THs as predisposing factors of MAFLD could be postulated. A potential vicious circle among these three conditions cannot be excluded. Identifying novel elements that may contribute to MAFLD could offer a practical approach to assessing children at risk of developing the condition.

## 1. Introduction

Non-alcoholic fatty liver disease (NAFLD) is one of the most common chronic liver diseases in the pediatric and adult population [1,2].

NAFLD is characterized progressively by steatosis, non-alcoholic steatohepatitis, and, subsequently, fibrosis.

There are many factors that cause NAFLD, and obesity is currently one of the main causes [3].

This disease has recently been renamed metabolic dysfunction-associated fatty liver disease (MAFLD) to underline the connection between NAFLD and metabolic dysfunction [4,5].

The diagnosis of NAFLD is based on an exclusion of other causes of steatosis while MAFLD diagnosis requires the concomitant finding of hepatic steatosis and metabolic dysfunction [6].

Metabolic dysregulation is characterized by at least two altered metabolic parameters (triglycerides, HDL, cholesterol levels, insulin resistance, and blood pressure) according to sex and age percentiles. However, the characterization of metabolic syndrome (MetS) in pediatric age is still a subject of debate [7,8].

An intricate relationship between the liver and the thyroid has been reported in both health and disease. The liver performs crucial functions in activating, inactivating, transporting, and metabolizing thyroid hormones (TH). THs influence the activities of hepatocytes and hepatic metabolism. The liver has a vital function in the processing of cholesterol and TG. Simultaneously, cholesterol and lipid metabolism, circulating lipoprotein levels, and intra-hepatic lipidic concentration are significantly influenced by THs. In particular, THs influence hepatic lipid balance through various pathways, such as promoting the delivery of free fatty acids to the liver for re-esterification into TG and enhancing fatty acid β-oxidation, consequently impacting hepatic fat accumulation [8]. The positive associations of thyroid function with the risk of hepatic steatosis suggest a plausible pathogenic role of THs on MAFLD.

There is a strong correlation between obesity and both MAFLD and thyroid dysfunction, in particular peripheral hypothyroidism. Obesity stands out as a primary contributor to the development of MAFLD, and, on the other hand, obesity influences thyroid function through a complex interplay of hormonal, metabolic, and inflammatory factors.

The aim of this narrative review is to highlight the relationship between MAFLD and thyroid function, particularly peripheral hypothyroidism, in children and adolescents with obesity in order to explore how THs act as predisposing factors in the onset, progression, and sustainability of MAFLD. Identifying novel elements that may contribute to MAFLD could offer a practical approach to assessing children at risk of developing the condition. Implementing early interventions has the potential to yield positive outcomes, emphasizing the importance of proactive measures in managing MAFLD.

## 2. Methods

A narrative review was presented; we performed a non-systematic summation and analysis of the available literature on the relationship between MAFLD and thyroid function in pediatric patients with obesity. In particular, unlike previous works, we have concentrated on examining the correlation between MAFLD and obesity, the relationship between thyroid hormones and obesity, and finally, the association between MAFLD and thyroid hormones in the pediatric population. Our aim is to elucidate these relationships despite limited scientific evidence in childhood.

**Figure 1:**
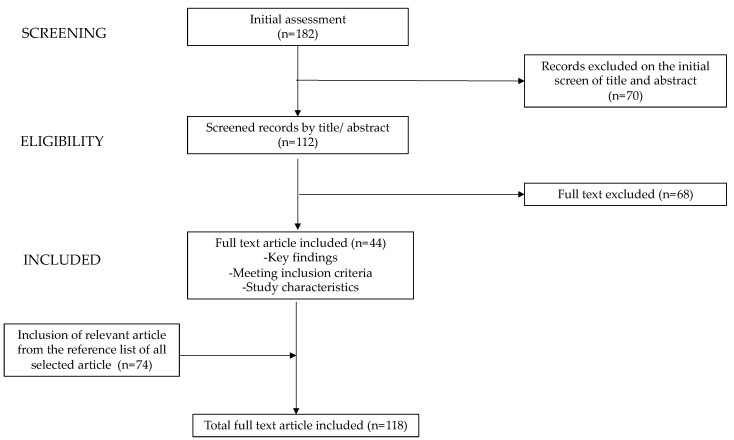
Process of paper selection and exclusion.

The electronic databases PubMed and Scopus were utilized for the review process. We employed keywords (alone or in combination) such as “MAFLD”, “thyroid”, “thyroid hormones”,“children”, “adolescents”, “obesity”, and “overweight”. The most relevant original manuscripts, reviews, and metanalysis published in English in the last 10 years were included. Case reports, case series, letters, and editorials were excluded. Starting from a total of papers (n = 182), the authors assessed the abstract (n = 112) and subsequently scrutinized full-text documents to discern studies of potential relevance (n = 44) within the literature. Additionally, the reference list of all articles was checked to identify relevant studies (n = 74). In Figure 1, the process of paper selection and exclusion is shown. The contributions were collected by S.T., CP., A.B., S.L., S.G.S, and A.Q. and critically analyzed with V.C. and I.D. The resulting draft was discussed and revised by V.C., I.D., and G.Z. The final version was then recirculated and approved by all.

## 3. Metabolic Dysfunction Associated with Fatty Liver Disease in Pediatric Obesity

### 3.1. Epidemiology

In recent years, the incidence of MAFLD has increased because it is related to the growth in the percentage of obesity in the world [9].

The growing incidence of obesity and NAFLD, in fact, is related to changes in food habits and sedentary lifestyles, especially during the SARS-CoV-2 pandemic [10].

According to Centers for Disease Control and Prevention (CDC) data, the highest prevalence of overweight and obesity is reported among teenagers aged 12–19 years (21.2%), (prevalence of 20.3% and 13.4% among 6–11-year-olds and 2–5-year-olds, respectively). In 2020, there were 39 million children under the age of 5 who were overweight or obese [11].

The global prevalence of NAFLD in the pediatric population has doubled in the last 20 years [12], with a global prevalence of 10%, 17% in teenagers, and 3% in 5–9-year-old children [1].

Obesity is the major cause of NAFLD. In fact, there is an increased prevalence rate of 20.23% in overweight and 38.47% in obese children and adolescents [13].

A meta-analysis by Anderson et al. highlighted that the prevalence of NAFLD in obese children was 34.2%, while in the general pediatric population was 7,6% [9].

Epidemiological data of MAFLD and NAFLD are not unique.

In some studies, the prevalence of MAFLD is 3–7% among children and adolescents [14,15].

MAFLD prevalence is 45% in child obesity clinics and 34% among overweight and obese children and adolescents (between 1 and 19 years) [14]

The incidence of MAFLD increased during the COVID-19 pandemic. In fact, quarantine measures and the closure of schools caused a reduction in physical activities and changes in food habits [15,16].

The prevalence of MAFLD is also influenced by ethnicity. In fact, white adolescents seem to be at higher risk than black adolescents. Geographical regions such as Central America and the Middle East have the highest prevalence of this disease [17,18].

The prevalence of MAFLD is twice as high in male children and adolescents than in females, according to data provided by the international literature. These results could be explained by different metabolic risk factors, adiposity, and body fat distribution [6,19].

### 3.2. Pathogenesis

MAFLD in children is believed to have a multifactorial origin, involving the intricate interplay of nutritional, hormonal, environmental, and genetic factors. In the context of obesity, hypertrophic adipose tissue releases pro-inflammatory cytokines, causing systemic insulin resistance [20]. Dysfunction in adipose tissue disrupts the balance of secreted adipokines, particularly leptin and adiponectin, which play pro- and anti-inflammatory roles, respectively [19]. In conjunction with intestinal dysbiosis and dietary intake, unregulated adipose tissue lipolysis results in elevated levels of free fatty acids. Consequently, there is a hepatic influx of free fatty acids, and hepatic de novo lipogenesis (DNL) further contributes to their accumulation, with subsequent options for oxidation, storage as triglycerides, or excretion via very low-density lipoprotein (VLDL) [20].

When the metabolic capacity of hepatocytes is surpassed, lipotoxicity occurs, and mitochondrial dysfunction triggers the excessive generation of ROS, while endoplasmic reticulum (ER) stress leads to the buildup of unfolded proteins. The cumulative effects expedite the progression to non-alcoholic steatohepatitis (NASH), characterized by hepatocyte inflammation, apoptosis, and fibrosis [19].

Environmental factors, including physical activity, socioeconomic factors, and dietary habits, play a significant role in the development of lipid disorders. Regular physical activity has been linked to a lower incidence of steatosis [21], while hypercaloric diets cause the accumulation of hepatic fatty acids (FA) with insulin resistance (IR) and increasing central adiposity [22]. Saturated fats facilitate FA oxidation through peroxisome proliferator-activated receptor alpha (PPARα) and de novo lipogenesis (DNL) in the liver [23]. An imbalanced omega-3/omega-6 ratio is also an important risk of non-alcoholic fatty liver disease (NAFLD) in children [24]. An increased intake of added sweeteners also causes MetS. In fact, high carbohydrate consumption leads to high blood glucose levels that activate the carbohydrate response element-binding protein (ChREBP), a key regulator of insulin-independent glycolysis and DNL [25]. Fructose metabolism, being insulin-independent, contributes to insulin resistance, elevated fasting glucose, insulin levels, and very low-density lipoprotein (VLDL) production [26,27]. Furthermore, excess fructose activates ChREBP and SREBP-1c [28], and high fructose intake may contribute to an increased prevalence of non-alcoholic steatohepatitis (NASH), hepatic fibrosis, and hepatocyte apoptosis [29]. Schwimmer et al. conducted a randomized study on boys aged 11–16 years old with NAFLD, revealing that those following a low-sugar diet (less than 3% of daily energy from added sugars) experienced an 8% reduction in hepatic fat content, compared to a 1% change in the control group consuming a typical diet. Additionally, transaminase levels dropped in the low-sugar diet group, with no significant differences in fasting insulin or triglycerides [30].

### 3.3. Diagnosis

Fatty liver disease secondary to metabolic conditions has been defined over the past decades as NAFLD. In pediatrics, the steatotic liver could also be detected in different conditions (such as viral hepatitis, Wilson disease, alfa1antitrypsin deficiency, metabolic disorders, coeliac disease, autoimmune hepatitis, and others), so that, in the past, diagnosis of NAFLD was based on the exclusion of other liver disorders [31,32].

In recent times, the transition from a negative definition (NAFLD) to a positive diagnosis, in the case of an association of liver steatosis with metabolic syndrome, has been proposed (MAFLD), underlining the strict relation to disease etiology and pathogenesis [6]. Even in the pediatric age, ESPGHAN encouraged this new definition, proposing an algorithm to avoid unnecessary investigations and costs to approach fatty liver disease [33].

For the diagnosis of MAFLD, the finding of steatosis via blood, imaging, or histological markers and the concomitant presence of metabolic dysregulation is important [5].

Metabolic dysregulation is characterized by at least two of these altered parameters according to sex and age percentiles: altered blood sugar, increase in triglycerides in the blood, increased waist circumference, hypertension, low serum HDL cholesterol concentrations, and a triglyceride-to-HDL cholesterol ratio of more than 2.25 [34].

Screening for steatosis is nowadays debated: which obese children to screen, at what age, and with which investigations are not universally accepted. ESPGHAN indicates screening with liver function tests and ultrasound in all obese children [31], while NASPGHAN recommends screening only via alanine aminotransferase (ALT) in all obese and overweight children with other risk factors at age 9–11 years [32]. The American Association for the Study of the Liver (AASLD) guidance instead does not suggest screening for other liver disorders in NAFLD in children with obesity [35].

For the detection of steatosis, according to Eslam M. et al., ultrasound and elevated ALT concentrations more than twice the upper limit of normal (<26 U/L for boys and <22 U/L for girls) are fundamental [36].

The combination of both ALT concentrations and ultrasound could be more favorable as ALT might be normal and US sensitivity decreases in children where hepatic fat accumulation remains below 30% [37,38,39].

The ALT level is an indicator of hepatocyte injury, but children with normal or not very high ALT concentrations may have liver histology abnormalities. In fact, a meta-analysis of 11 studies showed that 19% of people with NASH and 25% of people with NAFLD had normal ALT blood concentrations [40].

Different cutoff values of ALT are used in various studies for screening MAFLD. According to NASPGHAN, an elevation exceeding twice the upper normal levels should suggest further investigation. The upper normal level for males is 26 IU/L, while for females, it is 22 IU/L [32]. Schwimmer conducted studies with different cutoff values (ALT > 52 IU/L for females and >44 IU/L for males), as did Radetti (>40 IU/L), resulting in varying sensitivity (Schwimmer 88% and Radetti 54%) and specificity (Schwimmer 26% and Radetti 100%) for NAFLD [1,41]. Maffeis et al. found that children with NAFLD had mean ALT values of 42.04 IU/L, whereas children without NAFLD had mean values of 25.11 IU/L [42].

These studies demonstrate that the optimal cutoff of ALT is not straightforward, and it is important to use other parameters to improve the accuracy of screening.

CT is not indicated for diagnosis of steatosis due to radiation risk, while MRI and magnetic resonance spectroscopy, despite their accuracy for diagnosis, are only used in research studies due to their costs [43,44,45].

### 3.4. The Management and Treatment

Weight loss and lifestyle modifications have been shown to improve blood biomarkers of NAFLD. Lifestyle interventions, including low-calorie diets or engaging in physical activity, seem to offer benefits even in the absence of significant weight loss [46].

A sugar reduction, in particular in the meaning of fructose intake, seems to improve steatosis in obese adolescents [47]. The quality of fats ingested with n-3 PUFA and/or docosahexaenoic acid supplementation plays a pivotal role in the treatment of pediatric steatosis. However, the percentage of adult and pediatric patients capable of maintaining dietary changes for a long time is very low [48,49,50].

It is also important to take into account extra-hepatic comorbidities, such as insulin resistance and dyslipidemia, and adjustment of known risk factors. Treating children with obstructive sleep apnea (OSA) seems to enhance NAFLD severity, and improving vitamin D levels may reduce fibrosis [51,52]. Psychological support could also improve clinical outcomes because of the common association with psychological morbidity [53].

Since the definition of MAFLD has been only recently included in the literature and the pathogenesis of the two conditions (MAFLD and NAFLD) has significant overlap, no studies are currently available that compare the efficacy of the same molecule in a population of NAFLD patients compared to MAFLD. However, preliminary epidemiological studies have shown no differences in outcomes in the two forms [54,55,56].

Studies have explored metformin and vitamin E as potential treatments for pediatric steatosis. A randomized, placebo-controlled trial (TONIC trial) found no differences in levels of ALT after treatment with metformin or vitamin E and placebo. However, the group treated with those molecules did show evidence of reduced hepatocyte injury on liver biopsy [57]. While the effectiveness of antioxidants adjuvant vitamin E therapy has been demonstrated in adults with NAFLD, a comparable significant effect has not been evidenced in a meta-analysis based on pediatric patients [58]. Regarding the use of metformin, it may be beneficial in improving lipid parameters and regulating insulin metabolism in children with MAFLD; nevertheless, the results are encouraging but still controversial.

Docosahexaenoic acid, fish oil, oral insulin sensitizers, ursodeoxycholic acid, probiotics, and carnitine have all also been proposed as possible treatments for pediatric MAFLD; however, they have not proven to be very effective in treatment [59].

The use of probiotics holds potential benefits for pediatric NAFLD. Combining Lactobacillus acidophilus with other strains of Bifidobacterium or Lactobacillus appears to lower liver transaminases and enhance anthropometric, ultrasonographic, and lipidemic parameters when employed alongside lifestyle modifications. Other studies are necessary to validate these results [60].

Cysteamine bitartrate and polyunsaturated fatty acids (PUFAs), such as omega-3 fatty acids, also seem beneficial, but further research is warranted [61,62,63].

GLP-1 receptor agonists have also been studied for the treatment of MAFLD. Different molecules of this pharmacological class are studied; liraglutide seems to improve liver damage, favoring a reduction in liver steatosis, inflammation, and fibrosis. Studies also evidenced significant weight loss in patients treated with liraglutide due to the induction of delay in gastric emptying and increased satiation. However, studies on the efficacy and safety of GLP-1 receptor agonists in pediatric patients are limited, which is why those are not yet recommended for treating patients with NAFLD/MAFLD [64,65,66,67,68].

In some studies, Losartan is compared to a placebo in 8 to 17-year-olds with NAFLD. Losartan is important to reduce plasminogen activator inhibitor-1 (PAI-1), which is increased in children with NAFLD [69].

The elafibranor is studied in children from 8 to 17 years old with NASH; it seems to improve insulin sensitivity and induce the resolution of NASH in adult patients [70].

The prevalence of bariatric surgery among children and young individuals is on the rise globally [71].

While bariatric surgery can enhance histological findings in pediatric NASH, surgical intervention remains a topic of controversy except in exceptional clinical scenarios [72].

## 4. Thyroid Disorders in Children and Adolescents with Obesity

### 4.1. Pathogenic Mechanism

THs have a fundamental role in the regulation of metabolism by stimulating thermogenesis and fat oxidation and modulating food intake. Thyroid function is closely related to obesity, and the mechanism underlying this connection may function as an adaptive process to increase energy expenditure in an effort to mitigate additional weight gain. Specifically, the secretion of leptin, thyroid hormone resistance, and the inflammatory state associated with obesity have been identified as primary influential factors, also implicated in thyroid dysfunction.

Regarding leptin, it is constantly necessary in order to maintain the direct activation of the hypothalamus, resulting in the expression of the thyrotropin-releasing hormone (TRH) in the PVN. The TRH, in turn, stimulates the production of the TSH in the pituitary gland, which consequently stimulates the thyroid gland for the production of thyroid hormones [73,74]. This was also confirmed by a study carried out by Corica et al., which showed in pre-pubertal patients that the FT3/FT4 ratio (index of peripheral sensitivity) was positively associated with BMI (*p*  =  0.03) [75].

Additionally, fat tissue deiodinase is believed to contribute to the elevation of T3 levels, potentially serving as an adaptive process to enhance resting energy expenditure in individuals with excess weight or in response to the secretion of inflammatory cytokines by adipose tissue [76,77]. Furthermore, there have been reports of THs stimulating adipocytes, leading to heightened leptin secretion.

Concerning TH resistance, in children with obesity, a progressive reduction in T3 receptors in the pituitary gland leads to T3 resistance and, consequently, to an impaired negative feedback mechanism between thyroid hormones and the TSH. This mechanism inevitably causes an increase in TSH concentration. Apart from pituitary resistance, patients with obesity develop peripheral resistance to thyroid hormones, as suggested by Burman et al. [78]. In their study, the solubilized nuclear TH receptors in circulating cells decreased in obese children.

The increase in TSH concentration depends on the mechanism of T3 resistance and the secretion of several inflammatory cytokines by adipose tissue. Cytokines like interleukin-1 (IL-1), interleukin-6 (IL-6), and tumor necrosis factor-α (TNF-α) have been demonstrated to suppress iodide uptake by inhibiting sodium/iodide symporter (NIS) mRNA expression, both in Fisher rat thyroid cell line (FRTL-5) and in human thyrocytes [79].

These biological mechanisms, closely related to childhood obesity, may be responsible for changes in THs, such as a slight hyperthyrotropinaemia and a moderate increase in T3-FT3 hormones above the reference range or at the upper normal limits. The high concentrations of FT3-T3 lead to an increase in resting and total energy expenditure [75], including sleeping energy expenditure [80], thus contributing to reduced weight gain. T4-FT4 concentrations, instead, are not significantly different in obese children compared to normal-weight ones [79].

### 4.2. Clinical Evidence

Numerous hormonal abnormalities have been documented in overweight subjects. However, among these, thyroid dysfunction is frequently overlooked, particularly in pediatric patients.

The retrospective study by Stichel et al. [81], conducted on a cohort of 290 children with obesity and 280 healthy subjects, found elevated TSH levels (>4 mU/L), respectively, in 7.5% of children with obesity and 0.3% of controls. The medians of TSH and T3 levels were within the normal values but significantly higher in patients with obesity, while T4 levels did not differ.

The cross-sectional study by Reinehr et al. [75], carried out on 246 children with obesity and 71 normal-weight children, concluded that TSH and FT3 levels are significantly higher in patients with obesity compared with normal weight ones, whereas FT4 concentrations are similar in the two groups. Furthermore, the authors showed a significant correlation between weight loss and the reduction in TSH and FT3 levels, suggesting that the increase in these hormones seems to be a close consequence of obesity.

In a prospective study by Ghergherehchi and Hazhir [82], 190 overweight and 133 normal-weight children were evaluated, showing that TSH and total T4 concentrations were significantly higher in children with obesity compared with the control group. This study found a positive correlation between the BMI z-score and TSH and T4 levels. The incidence of thyroid antibodies instead was low among both groups. A significant correlation between fasting serum TSH and BMI SDS and waist-height ratio was also described by Dahal et al. [83].

Chen et al., in a cross-sectional study of adolescents between 12 and 18 years, reported a prevalence of 2.0% subclinical hypothyroidism, with median TSH levels higher in obese/overweight subjects compared to normal-weight ones [84]. Accordingly, Rumińska et al. showed that children with obesity had higher mean serum TSH levels compared to their lean peers [85].

Min An et al. analyzed thyroid dysfunction in Korean children with obesity, showing that the serum TSH level was associated positively with abdominal obesity and high-density lipoprotein cholesterol (HDL) and triglyceride (TG) levels, whereas the serum FT4 level was negatively correlated with abdominal obesity [86].

In addition to hormone abnormalities, some studies have focused on thyroid ultrasound changes observable in children with obesity. They described two different patterns: the increase in thyroid volume, which seems to be related to a slight elevation of TSH levels [75,87,88], and the presence of hypoechoic areas within the thyroid, similar to Hashimoto’s thyroiditis but without the presence of antithyroid autoantibodies [89]. These thyroid ultrasound abnormalities in patients with obesity may be caused by a low-grade inflammatory state, which is determined by an increase in inflammatory cytokines (IL-1, IL-6, and TNF-α) released from adipose tissue, which induces the vasodilatation and increased permeability of thyroid blood vessels and capillaries. After weight loss, reduced concentrations of inflammatory cytokines can normalize the thyroid volume and echogenicity at ultrasound [90,91].

Considering the important correlation between thyroid abnormalities and childhood obesity, a thyroid function screening in subjects with obesity must be considered. The European Society of Endocrinology Clinical Guideline on the Endocrine Work-up in Obesity, published in 2020, for example, suggests serum TSH assessment in all patients with obesity, not recommending routine assessment of fT3 because its levels in subjects with obesity depend on several factors which influence the peripheral conversion of thyroid hormones [92].

According to international guidelines, reference ranges of TSH levels for the treatment of hypothyroidism must be the same in patients with obesity and non-obese ones [93,94]. Overt hypothyroidism (i.e., TSH > 10 mU/L) must be treated, whereas the treatment of subclinical hypothyroidism (i.e., TSH 4.5–10 mU/L and T4 in range), nowadays is still controversial. European guidelines suggest treating isolated hyperthyrotropinemia only after considering several factors, such as the presence of thyroid autoantibodies or other causes of primary hypothyroidism [92]. The therapy with levothyroxine is only recommended in subjects with obesity and a diagnosis of hypothyroidism. The scientific literature agrees that weight loss can lead to a rapid reduction in TSH and fT3 concentrations, suggesting that thyroid abnormalities in obesity are reversible. This supports the recommendation of not routinely treating isolated hyperthyrotropinemia [95,96].

## 5. Association between NAFLD/MAFLD and Thyroid Function in Childhood Obesity

### 5.1. Pathogenic Mechanism

There is a correlation between overt or subclinical thyroid disease and NAFLD and the role of disthyroidism, and in particular of peripheral hypothyroidism, in the pathogenesis of NAFLD is supported by several data defining hypothyroidism-induced NAFLD [97].

The fundamental pathophysiology behind this correlation remains uncertain, though it likely involves numerous pathogenic mechanisms that are not necessarily mutually exclusive [98,99]. These mechanisms have been studied on animals and adult humans, but they might also apply to the pediatric population.

#### 5.1.1. Modulation of Glucose and Lipid Metabolism

THs play a crucial role in numerous physiological processes, primarily regulating basal energy expenditure through the modulation of glucose and lipid metabolism. THs affect almost every organ, and the liver is one of the most critical targets. These hormones enhance fatty acid production, alter insulin sensitivity within hepatic tissue, reduce hepatic gluconeogenesis, and promote lipid export and oxidation [100].

Peripheral hypothyroidism is characterized by metabolic modifications that are similar to MAFLD’s fundamental metabolic features. Besides signs of hyperlipidemia, individuals with hypothyroidism usually present decreased beta-oxidation of fatty acids and reduced hepatic release of triglycerides. Moreover, hypothyroidism might be linked to insulin resistance, a hallmark of MAFLD. Furthermore, hypothyroidism is correlated with heightened lipid peroxidation, which is a significant source of oxidative stress in liver parenchyma and contributes to liver cell damage [101,102].

Focusing on the pathophysiological linkage of hypothyroidism and MAFLD, knowing the capacity of THs to modulate lipid metabolism, hypothyroidism-induced NAFLD has commonly been imputed to decreased TH activity in the liver due to reduced TH blood levels, with a consequent decrease in lipid export and utilization, and a subsequent accumulation of fat in the liver [99].

#### 5.1.2. Intrahepatic Mechanism

However, other mechanisms have been identified in recent years. Some studies suggested there could be a reduction in intrahepatic TH concentration and/or signaling in individuals with NAFLD and that this localized primary hypothyroid condition diminishes hepatic lipase activity, which induces triglyceride storage. The reasons behind this resistance to thyroid hormone action within the liver are not fully understood. However, research suggests that intracellular fatty acids may hinder the activity of thyroid hormone receptors (THRs), establishing a vicious circle. Additionally, both mRNA and protein levels of type 1 iodothyronine deiodinase (DIO1), the enzyme responsible for converting T4 to T3 in the liver, seem highly sensitive to thyroid hormone serum levels. Consequently, decreased expression and activity of DIO1 might participate in intrahepatic hypothyroidism by impairing the transformation of T4 to T3 [103].

Furthermore, Ferrandino et al. showed that mildly decreased TH levels are associated with NAFLD, while, paradoxically, severely reduced TH values are not linked with NAFLD, demonstrating that the postulated intrahepatic mechanism is insufficient for explaining this condition and that extra-hepatic mechanisms might be involved. In their experiment, NAFLD occurred more frequently in mice with mild TH dysfunction, characterized by normal hepatic TH signaling and hepatic lipid consumption, than in severely hypothyroid mice with reduced TH signaling in their livers and strong inhibition of adipose tissue lipolysis, which decreases transportation of fat acids to the liver. According to these authors, the underlying explanation might be that low insulin secretion and adipose tissue insulin resistance impair the suppression of adipose tissue lipolysis, leading to increased delivery of fatty acids to the liver, where they are stored as triglycerides. Accumulation of lipids in the liver induces hepatic insulin resistance, impairing the suppression of hepatic gluconeogenesis and elevating blood glucose levels, thereby stimulating de novo lipogenesis in the liver and contributing to MAFLD [99].

#### 5.1.3. TSH-Related Effect

Recently, it has also been demonstrated that TSH itself, independently from TH blood levels, can lead to liver triglyceride accumulation, explaining subclinical hypothyroidism’s association with NAFLD. Kaltenbach et al. demonstrated in euthyroid children with severe obesity a significant correlation between high thyrotropin values and hepatic steatosis, an association that does not depend on age, gender, stage of puberty, BMI-SDS, and other risk markers for NAFLD. Higher TSH levels are related to a higher risk of NAFLD and a major grade of fatty accumulation observed in ecography [104]. By binding to the TSH receptor (TSHR) expressed on liver cells, TSH promotes hepatic SREBP-1c activity through the cAMP/PKA/PPARa pathway, concurrently reducing AMPK levels, thereby upregulating genes associated with lipogenesis [105]. Moreover, TSH increases hepatic gluconeogenesis by regulating gene transcription encoding rate-controlling enzymes. It boosts the expression of hepatic cAMP-regulated transcriptional coactivator-2, thereby activating glucose-6-phosphatase and cytosolic phosphoenolpyruvate carboxykinase. These enzymes facilitate the conversion of oxaloacetate to phosphoenolpyruvate and the hydrolysis of glucose-6-phosphate into free glucose and inorganic phosphate. The impaired modulation of carbohydrate metabolism due to heightened TSH values could potentially trigger reduced insulin sensitivity and type 2 diabetes, subsequently heightening the risk of MAFLD. Lastly, TSH may reduce the phosphorylation of HMGCoA reductase, leading to increased cholesterol levels (hypercholesterolemia) [106].

#### 5.1.4. Leptin, Fibroblast Growth Factor-21 Related Effects

Another element potentially contributing to the thyroid–liver relationship is leptin. Elevated in both hypothyroid and MAFLD patients, leptin promotes liver insulin resistance by dephosphorylating insulin receptor substrate-1, and it can promote hepatic fibrogenesis [107].

Recently, there has been a suggestion that Fibroblast Growth Factor-21 (FGF-21) plays a role in MAFLD. Among its various hormone-like functions, FGF-21 stimulates glucose absorption in adipose tissue, potentially enhancing glucose homeostasis. Many studies have described elevated blood FGF-21 levels in MAFLD, suggesting a potential FGF-21 resistance among these patients, although the specific mechanism remains to be elucidated [102].

#### 5.1.5. Oxidative Stress

Another pathogenetic theory involves oxidative stress. Free fatty acids (FFA) normally are subjected to β-oxidation, but in conditions of excessive accumulation of FFA in the liver cells, there is excessive oxidation of FFA, leading to the overproduction of reactive oxygen species (ROS). ROS stimulates lipid peroxidation that is linked to the activation of Kupffer cells and hepatic satellite cells, eventually leading to fibrosis. In patients with Hashimoto’s thyroiditis with hypothyroidism, elevated serum markers of oxidative stress have been found [107].

**Figure 2:**
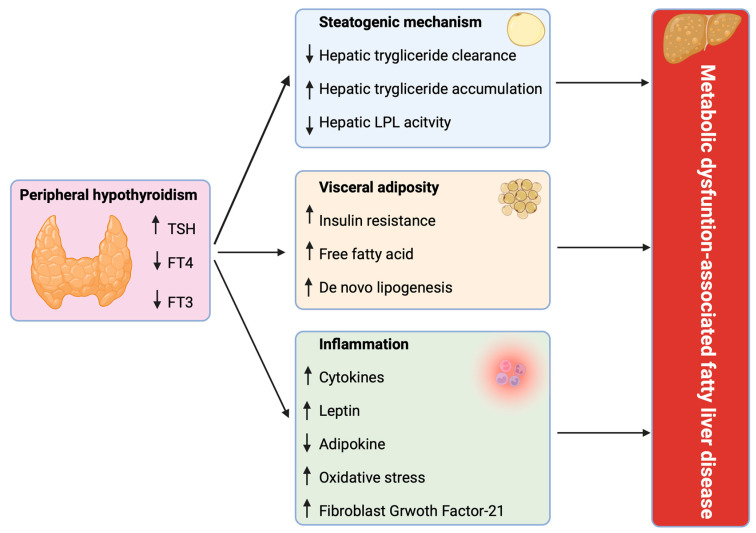
Pathogenic mechanism involved in the relationship between thyroid dysfunction and metabolic dysfunction–associated fatty liver disease (created with Biorender.com, accessed on 10 February 2024). TSH = thyroid-stimulating hormone; FT3 = free triiodothyronine; FT4 = free thyroxine; LPL = lipoprotein lipase.

In Figure 2, the potential pathogenic mechanisms involved in the relationship between thyroid dysfunction and MAFLD are schematized.

### 5.2. Clinical Evidence

Growing evidence links alteration of plasmatic TH levels, such as subclinical or overt hypothyroidism or hyperthyroidism, to the risk of developing a MAFLD [108].

As concerns about hypothyroidism, Chen et al. investigated the link between thyroid function and MAFLD. They found that both overt and subclinical hypothyroidism are seen as an independent risk factor for MAFLD in an adult population (OR = 1.27). Compared to those with normal thyroid function, individuals with subclinical hypothyroidism faced an elevated risk of all-cause and cardiovascular mortality in both the total population (hazard ratio [HR] for all-cause: 1.23; cardiovascular: 1.65) and the MAFLD population (HR for all-cause: 1.32; cardiovascular: 1.99) [109]. These data were also confirmed in the pediatric population, where Fan et al. found out that high plasmatic levels of TSH, even in overt hypothyroid states, are associated with the progression of liver fibrosis, worsening the prognosis of MAFLD pediatric patients. In addition, they also underline that high plasmatic levels of T3, FT3, TSH, and decreased FT4 correlate with an increased risk of developing overweight/obesity associated with MAFLD [110].

Even in an euthyroid population, Liu H. et al. found that MAFLD prevalence was 34.47%. This research study showed that the MAFLD group was characterized by a higher total triiodothyronine resistance index (TT3RI), thyroid feedback quantile-based index (TFQI)FT3, and FT3/FT4 levels in comparison with the non-MAFLD group, where TT3RI stands for fT3/TSH; TFQIFT3 stands for the thyroid feedback quantile-based index (TFQI)] = fT3 − (1-TSH), underlining the bond between hypothyroidism and MAFLD [111].

Concerning the prevalence of hyperthyroidism in subjects with MAFLD, the data in the literature are quite poor. In a large retrospective study from 2017 by Kaltenbach TE et al., in which they tried to prove the hypothesis that there is a connection between thyroid hormone levels and the risk of occurrence and severity of NAFLD in children and adolescents with obesity, hyperthyroidism was not found in any of the subjects [104]. In this study, a noteworthy correlation between elevated TSH levels in euthyroid youths with severe obesity and the development of hepatic steatosis was noted. This association remained significant, irrespective of factors such as age, gender, stage of puberty, BMI-SDS, and other risk markers for MAFLD.

Inizio modulo. Higher TSH levels were associated not only with a higher risk of MAFLD but also with an increased level of fatty infiltration detected in ultrasound scanning, underlining the possible significance of subclinical hypothyroidism as a predictive factor of metabolic comorbidity in children and adolescents with obesity.

The precise mechanism linking changes in THs plasma levels and MAFLD remains unclear, but it may involve dysregulation of mitochondrial homeostasis, endoplasmic reticulum stress, reduced insulin sensitivity, and chronic inflammation [112,113]. This last one is regarded as a pivotal factor in the pathogenesis of MAFLD and has the potential to contribute to the onset of MAFLD [114]. In this scene, studies revealed that low FT4 is related to higher levels of inflammatory biomarkers (e.g., tumor necrosis factor-alpha (TNF-α), interleukin-1, interleukin-6, and interferon-γ). Low-normal FT4 could also have a role in pro-inflammatory pathways and exacerbate liver fatty infiltration [115,116].

From the awareness of the relationship between altered thyroid functions and the risk of developing MALFLD comes the idea of using THs or THR agonists to prevent liver damage. The utilization of THs as therapeutic agents has been hindered for an extended period due to their lack of selectivity, leading to adverse side effects on various organs, including the heart and musculoskeletal system. Acknowledging that THRα primarily mediates these effects, the selective activation of THRβ is viewed as a promising strategy for developing new pharmacological treatments with a reduced side effect profile for different chronic liver diseases. In this scenario, a recent study by Caddeo A. et al. described the efficacy of a novel THRβ agonist called Resmetirom (TG68). Also known as MGL-3196, Resmetirom is a liver-directed THRβ agonist, orally administered, which has been demonstrated to lead to a significant reduction in liver triacylglycerides (TAGs), circulating LDL cholesterol and TAGs and to a higher rate of NASH resolution compared to a placebo. Both with a high dose (9.35 mg/kg/die) and with a low dose (2.35 mg/kg/die), TG68 leads to a reduction in liver volume and hepatic steatosis, enhanced liver injury, and levels of circulating TAGs and decreased intrahepatic lipid accumulation, demonstrated by histology test. All these effects were reached without the occurrence of undesirable toxic effects, primarily mediated through THRα [117].

In Table 1, we summarized selected manuscripts exploring the connection between disrupted thyroid functions and the risk of developing MAFLD.

## 6. Conclusions

MAFLD is a multisystem disorder characterized by the presence of fatty degeneration of the liver associated with overweight/obesity or prediabetes/T2DM or altered metabolism. In addition to being a constitutive element of MAFLD, obesity, particularly central obesity, is associated with various endocrine abnormalities, including thyroid dysfunction. THs are integral to the intricate balance of metabolic activities, ensuring energy homeostasis, and are indispensable for the optimal growth and development of the human body. Regarding liver homeostasis, THs have been suggested to interact with hepatic lipid homeostasis through different mechanisms, including stimulating the entry of FFA into the liver cells for esterification to TG and increasing mitochondrial β-oxidation of fatty acids to promote liver lipid accumulation. The literature data support a correlation between MAFLD and obesity, TH and obesity, and MAFLD and TH; however, results in the pediatric population are very limited. Even though the underlying pathogenic mechanism involved in the intricate relationship between MAFLD and thyroid function remains not fully elucidated, the role of THs as predisposing factors of MAFLD could be postulated in pediatric subjects with obesity. A potential vicious circle among these three conditions cannot be excluded.

Considering the pivotal role of thyroid function in the onset and progression of various diseases with significant implications for the healthcare system, such as MS, diabetes, and cardiovascular disease, integrating thyroid function monitoring into the management of young patients with MAFLD and/or at risk of MAFLD could greatly enhance comprehension of the interplay between these conditions. A comprehensive analysis of both TSH and TH release patterns is thus imperative to elucidate the significance of thyroid function at the intersection of several evolving fields, including the management of metabolic dysregulation.

Identifying TH as an additional modifiable factor that may contribute to predisposing and sustaining MAFLD could support prospective measures in preventing and managing this condition and improving outcomes.

Further studies into the correlation between MAFLD and thyroid function, particularly in pediatric patients with obesity, are crucial for advancing our comprehension and devising targeted strategies for risk management in this population.

## Figures and Tables

**Table 1:** Selected manuscripts on the connection between disrupted thyroid functions and the risk of developing MAFLD.

Reference	Study Type	Population	Main Result
Liu H, 2023 [111]	Cross-Sectional Study	6356 euthyroid adults, 2191/6356 with MAFLD	Elevated levels of FT3/FT4 and TFQIFT3 were significantly associated with the prevalence of MAFLD in euthyroid populations. The TyG index partially mediated the relationship between FT3/FT4, TFQIFT3, and MAFLD.
Chen CL, 2022 [108]	Retrospective Study	10,666 adults, 27.3% with MAFLD	Low thyroid function serves as an independent risk factor for MAFLD and is linked to an elevated risk of all-cause and cardiovascular mortality in the MAFLD population.
Fan H, 2022 [110]	Retrospective Study	18,427 adults: 661 MAFLD-diabetes, 3600 MAFLD-overweight/obesity, 691 metabolic disorder-MAFLD cases, 13,475 non-MAFLD controls)	Elevated levels of T3, FT3, and TSH, along with decreased FT4, were associated with an increased risk of overweight/obesity-MAFLD. Similarly, elevated T3, FT3, and decreased FT4 were associated with an increased risk of metabolic disorder-MAFLD.
Kaltenbach TE, 2017 [104]	Prospective cross-sectional study	332 overweight and obese children	Hepatic steatosis prevalence was 29.8%. Individuals diagnosed with NAFLD exhibited notably higher TSH levels compared to those without. Upon stratifying TSH values into quartiles, both univariate and multivariate analyses demonstrated a significant correlation with hepatic steatosis.
Caddeo A, 2021 [117]	In vitro study	Animal model	Treatment with TG68 led to a reduction in liver weight, hepatic steatosis, serum transaminases, and triglycerides.
